# Unmet Need for Contraception Among Female Sex Workers Initiating Oral Pre-Exposure Prophylaxis for HIV Prevention During Kenya's National Scale-Up: Results From a Programmatic Surveillance Study

**DOI:** 10.3389/fgwh.2021.747784

**Published:** 2022-02-21

**Authors:** Mercy Kamau, Abednego Musau, Daniel Were, Gladys Waruguru, Mark Kabue, Jane Mutegi, Marya Plotkin, Jason Reed

**Affiliations:** ^1^Jhpiego, Nairobi, Kenya; ^2^International Centre for Reproductive Health Kenya, Mombasa, Kenya; ^3^Jhpiego, Baltimore, MD, United States

**Keywords:** unmet need for contraception, female sex workers, oral PrEP, HIV prevention, scale-up, Kenya

## Abstract

**Background:**

Female sex workers (FSWs) experience a higher risk for both HIV acquisition and unwanted pregnancies compared to women in the general population. Pre-exposure prophylaxis (PrEP) for HIV prevention offers protection against HIV infection but has no contraceptive effect. We examined the determinants of unmet need for contraception among FSWs who initiated PrEP to inform programs and policies to optimize contraceptive services and avert unwanted pregnancy among this high-risk group.

**Materials and Methods:**

A cross-sectional analysis was conducted on routine, de-identified client data from a large-scale PrEP service delivery project, from February 2017 to December 2019. Data were collected from FSWs during clinic visits using Ministry of Health approved tools. Records for all 17,456 FSWs initiated on PrEP from 79 health facilities in 10 counties across three geographic clusters with high and medium HIV incidence were examined for eligibility for the analysis. Unmet need for non-barrier contraception was defined as not being pregnant, not currently using the non-barrier contraceptive method, and not trying to conceive or intending to have a child in the near future. Univariate and multivariable regression analyses were conducted with selected variables to examine associations.

**Results:**

In the 79 sites, eligible records from 17,063 FSWs who initiated PrEP were included. Two-thirds were under 30 years, and the majority were not married and had received PrEP at drop-in centers. Overall, the unmet need for non-barrier contraception was 52.6%, higher for those under 20 years of age (60.9%) and those served in public and private health facilities (67.4 and 83.2%, respectively) rather than drop-in centers (50.6%). Women from the Nairobi and Coast cluster regions reported a higher unmet need for contraception compared to those from the Lake region. All these associations were significant (*p* < 0.05) at the multivariate level.

**Conclusions:**

The high unmet need for non-barrier contraception among FSWs initiating PrEP highlights the need for integrated delivery of contraception services within PrEP programs. Identifying groups with a high unmet need could lead to higher success in an integrated program. Two recommended approaches include training healthcare providers to deliver clear contraception messaging during PrEP initiation and making a range of contraceptives accessible within PrEP services for high-risk groups. Furthermore, accelerated research on multipurpose prevention technologies is necessary to reduce the burden on individuals using multiple prevention products concurrently.

## Background

Female sex workers (FSWs) in low- and middle-income countries experience a triple burden of high HIV prevalence, high rates of sexually transmitted infections (STIs), and high risk for unintended pregnancy (1–3). Compared to adult women aged 15–49 years, FSWs are 21 times more likely to acquire HIV (1). The incidence of unintended pregnancies among FSWs approaches 30 per 100 person-years when evaluated through longitudinal studies (4). High rates of STIs among FSWs have also been documented (5, 6). Unintended pregnancies have far-reaching consequences for FSWs, including community stigma, income loss, and financial hardship from additional child raising expenses, which could culminate in dependency on sex work (7). FSWs who experience unintended pregnancies may seek abortions, often from non-professional providers (3, 7).

Kenya has made good progress in the fight against HIV, resulting in an estimated incidence–prevalence ratio under 3% in 2018 (1). This was achieved through the implementation of an aggressive strategy that prioritizes specific populations and geographic regions with high HIV prevalence and incidence (8). Kenya has a vibrant combination of HIV prevention programs that target key populations, with FSWs as a predominant priority population, and recent studies highlight significant progress in intervention uptake and the reduction of HIV prevalence among FSWs (9–11).

Globally, FSWs experience varying levels of unmet needs for modern contraceptives, and recent studies estimate that between 30 and 65% of FSWs have an unmet need for modern non-barrier contraceptive methods (7, 12–16). The use of condoms as a sole method for the prevention of HIV and pregnancy is suboptimal and influenced by retrogressive gender norms that preclude condom negotiation leading to low condom self-efficacy, and a high incidence of condom failure (17). Specific FSW segments, such as young sex workers, those with regular non-paying partners, FSWs who consistently use condoms, and those with children are associated with high unmet needs for non-barrier contraceptive methods (13, 15). However, these determinants are inconsistent among FSWs from different geographical and sociocultural contexts. Unmet contraceptive needs and their determinants in the context of emerging HIV prevention interventions, which overcome the drawbacks of condom use, are currently undocumented.

Unfortunately, uptake of non-barrier modern contraception by FSWs through current combination prevention programs in Kenya is suboptimal, which exposes them to unintended pregnancies (7). Studies in Kenya have described personal, community, and health system factors as impediments to contraceptive uptake among FSWs; these studies also suggested opportunities for program optimization (14, 18).

In 2016, Kenya introduced oral pre-exposure prophylaxis (PrEP) for HIV prevention using tenofovir-based regimens, adopting the September 2015 recommendations from the WHO. PrEP is delivered as part of combination HIV prevention strategies, which leverage existing platforms for HIV interventions (19). The use of PrEP helps counter persistent social and structural factors that limit FSWs' capacity to consistently use condoms to prevent HIV; these factors include gender norms, criminalization, intersecting stigma, and sexual and physical violence. If used consistently by eligible individuals, PrEP has the potential to drastically lessen HIV acquisition associated with condom non-use or condom breakage. However, there are concerns that PrEP use might culminate in unintended consequences such as increased STIs and unintended pregnancies, although the evidence is limited (3). The use of non-barrier contraceptives among FSWs using PrEP remains unknown and warrants investigation as PrEP scale-up continues to gather momentum in the sub-Saharan Africa region.

Kenya has made significant progress in promoting the uptake of PrEP and contributed to nearly half of all PrEP initiations in the sub-Saharan Africa region (20). FSWs form a large majority of the PrEP clients in Kenya's largest program (21). The non-barrier contraceptive use among FSWs who have initiated PrEP in programs in Kenya and determinants of use remain unknown. This study examines the unmet need for non-barrier contraception among FSWs initiated on PrEP and the determinants of their unmet need in one of Kenya's largest PrEP projects.

## Materials and Methods

### Study Setting

**Figure 1:**
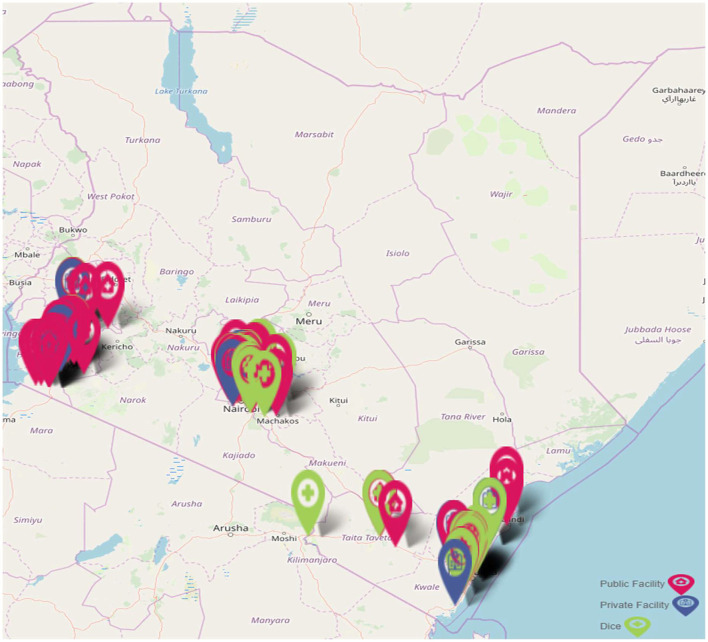
Jilinde project implementation sites.

We conducted this study within the context of the Jilinde project, a 5-year project that began implementation in 2016 in three Kenyan cluster regions (Coast, Lake, and Nairobi), these clusters comprise of 10 counties (22). Jilinde has been supporting PrEP delivery in 93 sites consisting of 45 government-run health facilities, 12 private health facilities, and 36 drop-in centers (DICEs). The distribution of the sites is summarized in Figure 1.

These sites deliver PrEP to any individuals at substantial, ongoing risk for HIV infection, such as self-identifying FSWs, men who have sex with men, adolescent girls, and young women, and HIV-negative individuals in serodiscordant relationships. This study is focused on data from the FSW PrEP clients.

### Data Collection and Sources

Data were collected between February 2017 and December 2019 from 79 facilities (34 DICEs, 37 public, and 8 private) that served FSWs. Data collection was done by healthcare providers during the PrEP initiation visit, the 1-month follow-up visit, and, subsequently, during monitoring visits scheduled for every 3 months. The main data source for this study was the PrEP Client Encounter Form (CEF), which is a nationally approved client record employed to capture clinical data related to all PrEP-related encounters in the country. At the initiation visit, providers use the CEF to capture sociodemographic details, responses to 10 behavioral risk questions, reproductive health questions, and clinical assessment and management information. Responses for the sociodemographic, behavioral risk, and reproductive questions are captured by filling in content or applying a tick mark to the checkbox alongside each question to imply an endorsement from a client, otherwise, the checkbox is left blank. During the monitoring visits, providers capture only selected variables. The Jilinde project employs an end-to-end encrypted Android application to facilitate the entry of anonymized client information from the CEF. Data are transmitted in real-time to a centralized and secure Microsoft SQL server owned by the project. Data are routinely extracted, cleaned, and analyzed to generate insights that are employed for the implementation of quality improvement. Data quality assurance audits and supportive supervision were employed to enforce data quality.

### Data Analysis

The study adopted a retrospective cross-sectional analysis of de-identified data from the initiation visits as captured by the CEF. All the records for FSW clients who accessed PrEP through Jilinde-supported sites were captured and examined for inclusion in the study. Data from FSWs who were of reproductive age (15–49 years) were included. Data from FSWs who were older than 49 years, were pregnant, reported having had a hysterectomy, or were trying to conceive were excluded. Unmet need for contraception was defined as being in reproductive age, not being pregnant, not using non-barrier contraception, and not trying to conceive or not planning to do so in the near future. All FSWs were deemed to be sexually active by reporting at least one sexual partner in the month preceding the PrEP initiation visit. Analysis was conducted using IBM SPSS 25.0 (IBM Corp., Armonk, NY, USA). Initially, data were summarized using proportions, means, interquartile range, and medians. Univariate logistic regression models were constructed to identify an association between demographic variables (age, marital status, and referral channel) and selected behavioral risk questions with unmet need for contraception as the outcome variable. Furthermore, variables that illustrated a significant relationship at univariate analysis were included in a multivariable logistic regression model to determine the independence of the associations. Associations were summarized using (crude) unadjusted odds ratio (COR), adjusted odds ratio (AOR), and their 95% CI. A *p*-value < 0.05 was considered statistically significant. Results of the regression analyses were summarized in tables.

### Ethical Considerations

Ethical approvals for this study were obtained from the Kenya Medical Research Institute (KEMRI) Scientific Ethics Research Unit (Non KEMRI 601) and the Johns Hopkins Bloomberg School of Public Health institutional review board (IRB 8346). Confidentiality standards stipulated by the Ministry of Health were adhered to when handling the data.

## Results

**Figure 2:**
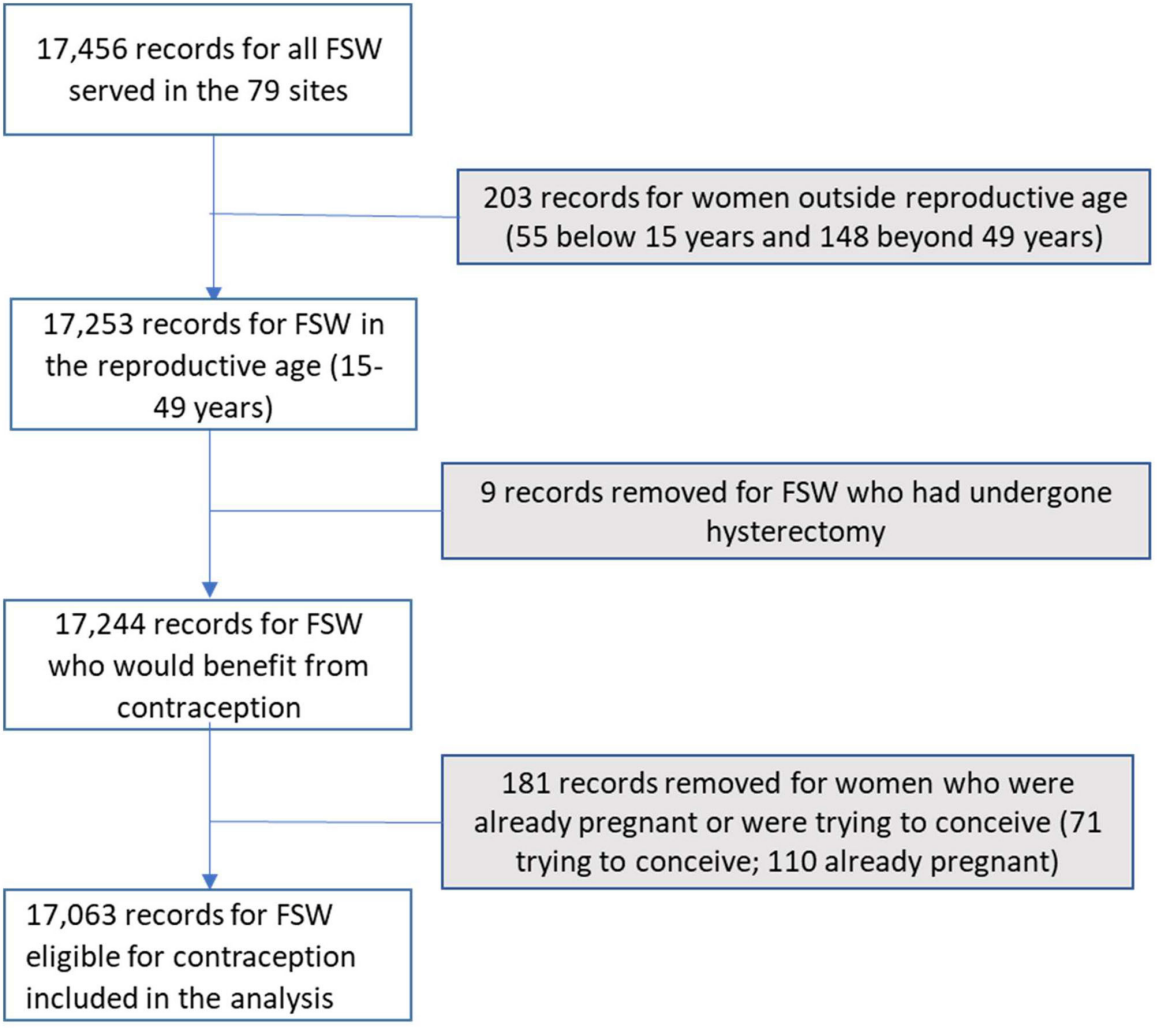
Flow diagram for the number of female sex worker (FSW) records processed.

Between February 2017 and December 2019, 17,063 FSWs eligible for contraceptives were initiated on oral PrEP in the 79 sites as depicted in Figure 2. Nearly all (91.6%) of the FSWs were older than 20 years. The mean age for the FSWs was 27.3 years (SD 6.6 years). The majority of the FSWs (91.3%) were not currently married and 90% received PrEP from DICEs. The FSWs reported an average of five sexual partners within the 30 days preceding the PrEP initiation. Table 1 summarizes the social, demographic, and behavioral characteristics of the FSWs.

**Table 1:** Associations between unmet contraception needs and independent variables at univariable and multivariable regression analysis.

**Variables and categories**	**Satisfied criteria for unmet contraception need** ***N*** **(%)**		**COR (95% CI)**	***p*-value**	**AOR (95% CI)**	***p*-value**
Unmet need	Yes (*n* = 8,980; 52.6%)	No (*n* = 8,083; 47.4%)				
**Cluster region**						
Coast	2,818 (70.0%)	1,206 (30.0%)	1.95 (1.81–2.11)	<0.001	1.70 (1.56–1.85)	<0.001
Lake	777 (24.7%)	2,375 (75.3%)	0.27 (0.25–0.30)	<0.001	0.22 (0.02–0.24)	<0.001
Nairobi	5,385 (54.5%)	4,502 (45.5%)	Ref.		Ref	
**Age in years**						
15–19	877 (60.9%)	564 (39.1%)	1.66 (1.47–1.87)	<0.001	1.55 (1.36–1.76)	<0.001
20–24	2,930 (54.9%)	2,406 (45.1%)	1.30 (1.20–1.40)	<0.001	1.29 (1.19–1.40)	<0.001
25–29	2,435 (52.6%)	2,193 (47.4%)	1.18 (1.10–1.28)	<0.001	1.19 (1.10–1.30)	<0.001
30 or more	2,738 (48.4%)	2,920 (51.6%)	Ref.		Ref	
**Marital status**						
Currently married	738 (49.5%)	753 (50.5%)	0.87 (0.78–0.97)	0.011	0.92 (0.82–1.03)	0.162
Not currently married	8,242 (52.9%)	7,330 (47.1%)	Ref.		Ref.	
**Health facility type**						
Drop-in center	7,775 (50.6%)	7,602 (49.4%)	0.49 (0.44–0.56)	<0.001	0.38 (0.34–0.44)	<0.001
Private facility	361 (83.2%)	73 (16.8%)	2.39 (1.81–3.16)	<0.001	0.91 (0.67–1.24)	0.565
Public facility	844 (67.4%)	408 (32.6%)	Ref.		Ref.	
**Referral channel**						
Peer referral	3,439 (60.5%)	2,242 (39.5%)	1.38 (1.28–1.49)	<0.001	1.00 (0.91–1.09)	0.981
Within facility referral	2,684 (45.0%)	3,274 (55.0%)	0.74 (0.68–0.79)	<0.001	0.49 (0.45–0.54)	<0.001
Community referral	2,857 (52.7%)	2,567 (47.3%)	Ref.		Ref.	
**HIV-positive partner**						
No	8,824 (52.5%)	7,971 (47.5%)	0.79 (0.62–1.02)	0.066	–	
Yes	156 (58.2%)	112 (41.8%)	Ref.		–	
Experiencing IPV/GBV*						
No	8,847 (52.8%)	7,904 (47.2%)	1.79 (1.40–2.27)	<0.001	1.99 (1.54–2.59)	<0.001
Yes	109 (38.5%)	174 (61.5%)	Ref.		Ref	
Recent STI*						
No	8,332 (53.0%)	7,376 (47.0%)	1.27 (1.14–1.42)	<0.001	1.26 (1.12–1.43)	<0.001
Yes	624 (47.1%)	702 (52.9%)	Ref.		Ref	
Recurrent PEP use*						
No	8,669 (53.2%)	7,636 (46.8%)	1.75 (1.50–2.03)	<0.001	1.65 (1.40–1.95)	<0.001
Yes	287 (39.4%)	7,636 (46.8%)	Ref.		Ref	
**Sex under influence of alcohol or drugs**						
No	6,046 (54.2%)	5,099 (45.8%)	1.21 (1.13–1.28)	<0.001	1.09 (1.02–1.17)	0.012
Yes	2,934 (49.6%)	2,984 (50.4%)	Ref.		Ref	
**Condom use consistency**						
Consistent use	386 (71.0%)	158 (29.0%)	2.25 (1.87–2.72)	<0.001	1.48 (1.21–1.81)	<0.001
Inconsistent use	8,594 (52.0%)	7,925 (48.0%)	Ref.		Ref	

*COR, crude odds ratio; AOR, adjusted odds ratio; IPV/GBV, intimate-partner violence/gender-based violence; STI, sexually transmitted infection, PEP, post-exposure prophylaxis.*

^*^Missing records.

**Figure 3:**
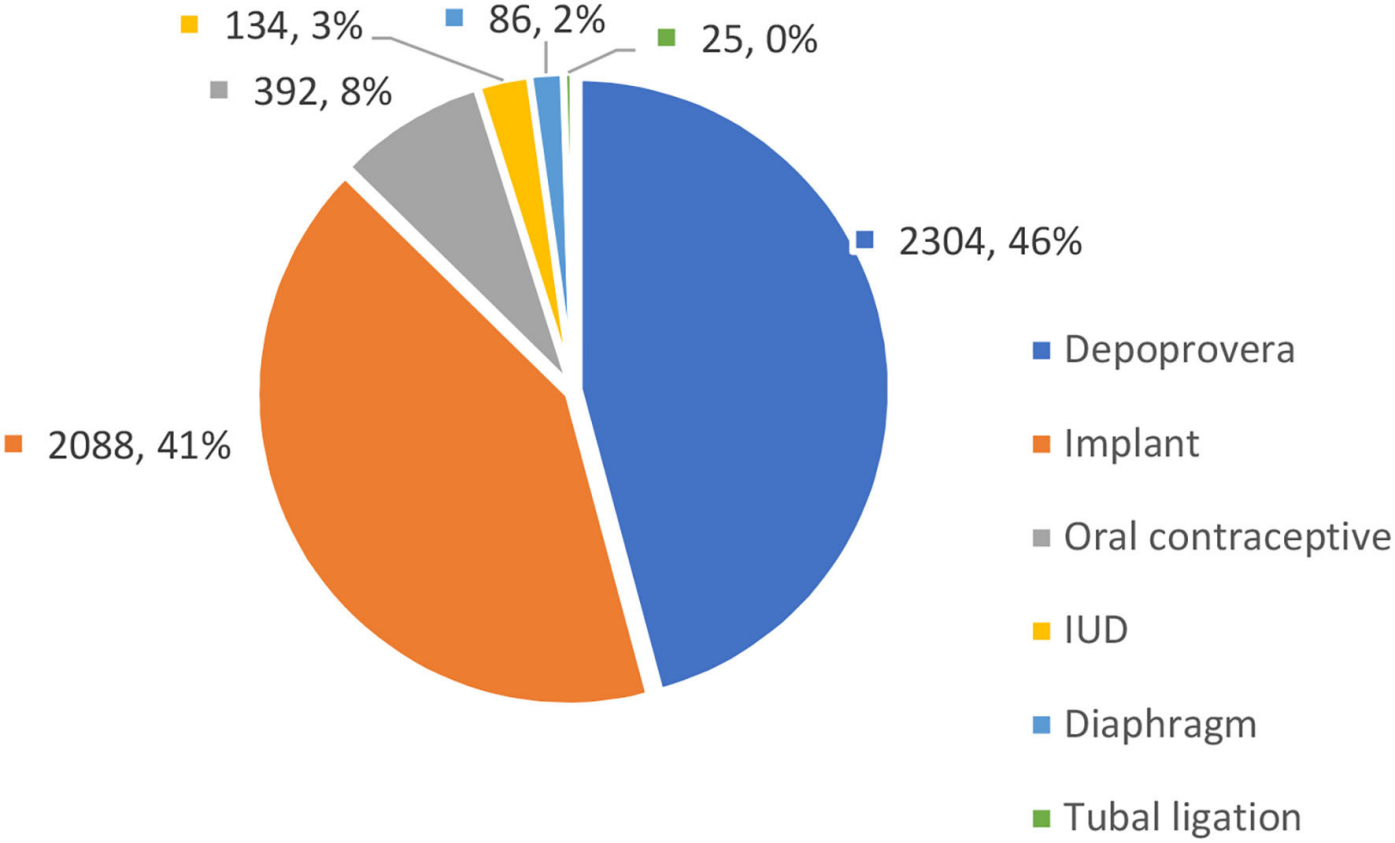
Method mix for non-barrier contraceptive methods among female sex workers (FSWs) (*n* = 4,392).

Slightly over half (52.6%) of the FSWs initiating oral PrEP reported that they had unmet contraception needs. The non-barrier contraceptive method mix for the FSWs are summarized in Figure 3.

In univariate logistic regression analysis, social demographic factors that included age, geographical location, and marital status were associated with unmet contraception needs. Where FSWs received PrEP services and referral channels were also associated with unmet contraception needs. Younger FSWs, those from the Coastal region, and those receiving PrEP from public facilities were more likely to report unmet need for contraception compared to those who were older, from Nairobi or Lake regions, and receiving services from DICEs. These results are summarized in Table 1. Furthermore, when examining HIV risk behaviors, FSWs not reporting intimate or gender-based violence, without a recent STI, not having sex under the influence of alcohol or recreational drugs, and using condoms consistently were associated with higher unmet contraception needs.

In multivariable analysis, being a younger FSW was associated with a higher unmet need for contraception (as shown in Table 1). The study revealed that FSWs aged 15–19 years (AOR = 1.55; CI = 1.36–1.76), 20–24 years (AOR = 1.29; CI = 1.19–1.40), and those aged 25–29 years (AOR = 1.19; CI = 1.10–1.30) experienced a higher unmet need compared to their counterparts 30 years or older.

The FSWs who received PrEP via DICEs reported lower unmet needs for contraception (AOR = 0.38; CI = 0.34–0.44) compared to FSWs who were served in public and private health facilities. Additionally, FSWs who had not experienced intimate-partner or gender-based violence (AOR = 1.99; CI = 1.54–2.59), those not reporting recent STIs (AOR = 1.26; CI = 1.12–1.43), or those had not recurrently taken post-exposure prophylaxis (PEP) for HIV (AOR = 1.65; CI = 1.40–1.95), FSWs who did not have sex under the influence of alcohol or recreational drugs, or who consistently used condoms reported higher unmet needs. Lastly, FSWs in the Lake Victoria region had lower unmet needs (AOR = 0.22; CI = 0.20–0.24). All these associations were significant (*p* < 0.05) at the multivariable level and are summarized in Table 1.

## Discussion

In this study, the unmet need for contraception was present in more than half of FSWs who did not intend to conceive or have a child in the near future. Nearly all FSWs were not using condoms consistently and required PrEP for HIV prevention to fill this void, given the high HIV prevalence (29.3%) among FSWs in Kenya. Although PrEP is highly efficacious in preventing HIV infection, grants control to the FSWs, and thus overcomes the need for cooperation from their sexual partners to use condoms for HIV prevention, it does not prevent unwanted pregnancies (3). With the trend observed in this study, FSWs initiating PrEP for HIV prevention have a concurrent vulnerability to unintended pregnancies, which is not comprehensively addressed through the existing implementation models in Kenya. These findings underscore the importance of strengthening the integration of voluntary contraception services and counseling within current PrEP and broader FSW HIV prevention programs (14).

The findings on unmet needs for non-barrier contraception among FSWs initiating PrEP from this study are not different from other studies conducted among FSWs despite the milestones attained in establishing comprehensive sexual and reproductive health programs in the sub-Saharan African region. Recent studies conducted in Côte d'Ivoire, Cameroon, Kenya, Madagascar, and Malawi have highlighted that only 25–55% of FSWs were using a non-barrier contraceptive method (14–16, 23, 24). In these studies, over half of FSWs reported experiencing an unwanted pregnancy in their lifetime. Furthermore, the use of long-acting reversible contraceptive methods, which are associated with lower failure rates, has been suboptimal among FSWs who use contraception (25). Since FSWs are a priority target population for HIV prevention programs, these findings imply that current programs do not comprehensively meet the sexual and reproductive health needs of FSWs. Additionally, barriers to contraceptive use for FSWs that warrant the attention of PrEP programs have also been uncovered through qualitative studies. These barriers include unsupportive clinic infrastructure, such as long wait times, inconvenient operating hours, and perceived compulsory HIV testing; discriminatory provider-client interactions; negative partner influences; laws that criminalize sex work, among others (7, 18, 25, 26).

Age was a significant predictor of unmet contraceptive needs. Younger FSWs (15–24 years) had an increased likelihood of unmet contraceptive need compared to their older counterparts. These findings concur with findings elsewhere that found low contraceptive use among non-PrEP-seeking FSWs within this age group (16, 27, 28). Compared to older FSWs, younger FSWs, especially those in adolescence, experience poor access to HIV prevention and contraceptive services, have limited knowledge, and are more vulnerable to HIV transmission, unwanted pregnancies, and violence (29). In recognition of the unique needs of young key populations, including FSWs, in 2018, Kenya developed *National Implementation Guidelines for HIV and STI Programming for Young Key Populations* (19). Within the context of PrEP scale-up in Kenya, the operationalization of these guidelines presents an opportunity for implementers to ensure that the contraceptive needs of younger FSWs are adequately addressed.

The type of facility through which FSWs accessed PrEP services emerged as a predictor of unmet need for contraceptive use. To widen coverage, Kenya has integrated PrEP into all channels that potential beneficiaries can access. Most FSWs initiating PrEP in Kenya do so through DICEs, which provide stigma-reduced and comprehensive HIV combination prevention services to self-identifying FSWs through in-clinic and outreach delivery (21). This study found that FSWs initiating PrEP through DICEs had lower unmet contraception needs compared to those initiating through public and private health facilities. DICEs offer an expanded prevention package as a one-stop shop that includes sexual and reproductive health services, including STI and cancer screening and treatment, contraceptive services, and peer education. On the contrary, private and public facilities attract non-identifying FSWs and often serve clients through multiple service delivery points, each offering unique services; clients are referred between multiple points if they need multiple services. The fragmented nature of services acts as a disincentive for FSWs to access multiple services since they suffer income losses from long wait times compounded by stigma and discrimination from providers and other clients (30). Sensitivity training for providers and streamlining services to increase efficiency in these facilities could enhance contraceptive use among FSWs.

Finally, unmet contraceptive need was associated with the absence of certain HIV risk behaviors among FSWs including reporting violence, recent STI, having sex under the influence of alcohol and drugs, and recurrent PEP use. These findings contrast with most studies that have established that FSWs experience concurrent vulnerabilities and challenges when accessing HIV prevention services and violence mitigation measures. For instance, women who experience violence are more likely to use condoms inconsistently, and this might extend to the use of contraceptives and PrEP (13). In our study, FSWs who had not experienced intimate-partner or gender-based violence, had not reported recent STI, or had not recurrently taken PEP for HIV, and those who did not have sex under influence of alcohol or recreational drugs reported higher unmet needs. We hypothesize that FSWs who were not prone to violence, STIs, or circumstances where the recurrent use of PEP was needed had a lower HIV and pregnancy risk perception and were, therefore, less likely to consistently engage with the existing comprehensive prevention services through DICEs. In the same vein, inconsistent condom use was associated with a lower unmet need for non-barrier contraceptive methods. A higher vulnerability among FSWs has been associated with increased familiarity with and access to prevention services when such services exist (31). In our case study, DICEs, which served over 90% of clients, implement robust violence mitigation and response interventions and programmatic data from Kenya highlights good outcomes (32). Access to violence-related interventions, STI treatment, PEP for HIV, and counseling for harmful alcohol use might have presented FSWs with opportunities to benefit from auxiliary services including contraceptives. We believe that FSWs with low-risk perceptions presenting for PrEP will benefit from provider messages that reinforce the adoption of complementary prevention interventions including contraceptives. This further argues for the need for continued implementation of an expanded package of services for FSWs inclusive of robust structural components to synergize prevention. To increase adherence and reduce the burden on individuals using multiple prevention products concurrently, research currently underway on multipurpose prevention technologies (MPTs) that prevent HIV, STIs, and pregnancy will need to be accelerated. Until when MPTs will be available, efforts to increase access to PrEP and contraception may leave FSWs vulnerable to STIs, and programs will need to strengthen STI diagnosis and treatment services.

Finally, this study was conducted using routinely collected program data. Gathering rich client-level data as part of routine implementation can generate useful insights that can facilitate the delivery of tailored intervention components to beneficiaries who possess unique characteristics. We encourage programs to use digital data systems to collect longitudinal data to generate trends on the adoption of contraceptive methods and other interventions. Insights from analyzing these data could inform programmatic course corrections and accelerate the attainment of prevention outcomes.

## Limitations

This study is not devoid of limitations. First, the data analyzed were collected within the context of a routine program and not within a controlled study setting. A national tool was used for capturing the data for the variables employed in the study and data collected by providers during the course of delivering clinical services. This limited the number of variables included in the study. However, this approach facilitated the gathering of data from more FSW clients than would be attained through a research study. Second, data from this study was collected through self-reports during face-to-face interactions between providers and their clients. Self-reporting is subject to social desirability bias. It is possible that FSW clients might have over-reported certain characteristics and behaviors to increase their chances of being initiated on PrEP. We do not suspect that this was a major issue since no incentives were provided to clients, and clients were already receiving complementary services in the same health facilities. Third, there is a potential that providers erred when capturing data due to the high workload from serving many clients. However, the magnitude of these errors is likely to be minimal since quarterly, site-level, data quality audits were conducted to ensure data accuracy. Finally, data are reported for FSWs from 10 out of the 47 counties in Kenya within selected health facilities. Therefore, the study results cannot be generalized to all FSWs on PrEP across the whole country. However, these 10 counties are among the 19 counties earmarked for intensive HIV prevention in Kenya and originate the largest population of key populations including FSWs in Kenya. The findings of this study provide a foundation on which future studies with robust recruitment methodologies can be conducted.

## Conclusions

The findings of this study indicate that FSWs initiating PrEP from various service delivery models have high unmet contraception needs. Unmet needs varied across implementation geographies, the service delivery model, age, and specific HIV transmission risk behaviors. Furthermore, the findings highlight the need for integration of contraception services with PrEP and other HIV prevention services, right from messaging to PrEP initiation and during every subsequent clinical visit. Programs should put more effort into training and empowering healthcare providers offering PrEP to identify FSWs with unmet contraception needs, create promotion messages to increase demand for both PrEP and contraception services, and make contraceptives accessible and acceptable to FSWs. PrEP programs should also strive to address the structural and health system barriers that impede uptake and continuity of contraceptive and PrEP services. These efforts would go a long way in concomitantly preventing unintended pregnancies and HIV infections among FSWs.

## Data Availability Statement

The original contributions presented in the study are included in the article/supplementary material, further inquiries can be directed to the corresponding author/s.

## Author Contributions

MKam: conceptualization, investigation, methodology, data analysis, validation, visualization, writing—original draft, and writing—review and editing. AM and GW: conceptualization, methodology, supervision, investigation, and writing—review and editing. DW: conceptualization, investigation, project administration, funding acquisition, visualization, methodology, supervision, writing—original draft preparation, and writing—review and editing. JM: conceptualization, investigation, methodology, data analysis, validation, writing—original draft preparation, and writing—review and editing. MP: conceptualization, validation, investigation, writing—original draft preparation, and writing—review and editing. JR: conceptualization, validation, writing—original draft preparation, and writing—review and editing. All authors contributed to the article and approved the submitted version.

## Funding

This work was supported, in whole, by the Bill & Melinda Gates Foundation [INV-007340] through Jhpiego.

## Conflict of Interest

MKam, AM, DW, and JM were employed by Jhpiego, Nairobi, Kenya. MKab, MP, and JR were employed by Jhpiego, Baltimore, MD, United States. The remaining author declares that the research was conducted in the absence of any commercial or financial relationships that could be construed as a potential conflict of interest.

## Publisher's Note

All claims expressed in this article are solely those of the authors and do not necessarily represent those of their affiliated organizations, or those of the publisher, the editors and the reviewers. Any product that may be evaluated in this article, or claim that may be made by its manufacturer, is not guaranteed or endorsed by the publisher.
